# A Novel Pneumatic Balloon Saved the Lives of Three Patients With Refractory Pelvic Hemorrhage After Peripartum Hysterectomy

**DOI:** 10.7759/cureus.85049

**Published:** 2025-05-29

**Authors:** Ali M El Saman, Hossam O Hamed

**Affiliations:** 1 Department of Obstetrics and Gynecology, Assiut University, Assiut, EGY; 2 Department of Obstetrics and Gynecology, Qassim University, Burraidah, SAU

**Keywords:** life-saving procedures, novel technique, pelvic hemorrhage, peripartum hysterectomy, pneumatic balloon

## Abstract

This case report discusses three patients who presented in our obstetric emergency with refractory pelvic hemorrhage following cesarean hysterectomy due to placenta accreta. They were referred from district hospitals with pelvic towel packing as a lifesaving procedure after failure of pelvic angiographic embolization or internal iliac artery ligation. After stabilization, we did a relaparotomy to remove the surgical towels, which led to the recurrence of excessive pelvic hemorrhage. The new pneumatic balloon was prepared from two surgical rubber gloves inserted into each other and ligated around a plastic catheter. The gloves were placed inside the pelvis and then inflated by connecting the air pump of a sphygmomanometer to the catheter outlet. A tight external abdominal binder was applied to keep the balloon in place. The bleeding stopped in all cases when the balloon pressure was calibrated at 40-60 mmHg and kept for 24-72 hours. The balloon was then gradually deflated and removed in a bedside procedure. This pneumatic balloon is a feasible and lifesaving procedure. It can be an alternative emergency tool to towel packing when other methods fail, in terms of the advantages of bedside removal. Large studies are essential to evaluate its efficacy and safety and to estimate the optimum pressure.

## Introduction

Cesarean hysterectomy is a lifesaving procedure in atonic postpartum hemorrhage, uterine rupture, placenta accreta, and other indications [1-3]. Post-hysterectomy pelvic hemorrhage can occur even with secure closures of all pedicles because of excessive tissue trauma in a ruptured uterus, coagulopathy, or infection. Multiple surgical interventions, including hypogastric vessel ligation or embolization [4] and pelvic compression methods [5-9], were studied. 

Pelvic compression is a rapid, lifesaving method that does not need much surgical experience. In the literature, it was performed either by packing using a surgical towel [5-7] or by using fluid-filled balloons [8,9] with good results. The main disadvantages of surgical towels are the need for relaparotomy for towel removal, in addition to the theoretical high risk of infection and tissue trauma due to their braided texture. On the other hand, few studies have studied compression using inflated balloons. The first one is dated to 1926, when Logothetopulos described his technique, which was later retitled as a mushroom, umbrella, or parachute and known as the Logothetopulos pack [10]. 

More recently, different types of balloons have been studied for the same purpose after cesarean hysterectomy, including the Bakri balloon in a case series that included 3 patients [9], and a condom was inflated by an attached Foley’s catheter in a case report with reported good results [8]. The main advantage of fluid-filled balloons is the ability to drain and then remove them without relaparotomy. The Bakri balloon has a limited stated capacity of 500 mL of fluid as issued by the manufacturer [11], which may not be enough to tamponade a wide, oozy pelvic surface area. 

To the best of our knowledge, there is no peer-reviewed published data studying pelvic pneumatic balloons in the management of refractory obstetric pelvic hemorrhage. In this case series, we presented a novel simple pneumatic balloon that was used as a lifesaving tool in three patients who presented with refractory hemorrhage following cesarean hysterectomy after failure of other surgical methods.

## Case presentation

The current case series included three patients with intractable pelvic hemorrhages after postpartum hysterectomy. They were referred to the obstetric emergency department in the women's health hospital, affiliated with Assiut University Medical School. The indication of cesarean subtotal hysterectomy in all cases was placenta previa of a major degree, which was complicated by placenta accreta that was first discovered during cesarean section. Table 1 shows the complete clinical and lab patient data at admission. All cases had previous C-section (CS) deliveries. Lab investigations showed anemia, leukocytosis, low platelet count, and high C-reactive protein. Table 1 also showed the surgical data and interventions which have performed to control bleeding prior to their arrival at our obstetric emergency.

**Table 1:** Patients’ clinical, laboratory, and surgical data at admission

Patient data at admission	First case	Second case	Third case
Age	34	38	37
Parity	P4+1	P5+0	P5+2
Time since delivery (days)	2	3	4
Blood pressure (mm/Hg)	90/50	100/60	90/60
Pulse (b/min)	130	115	110
Pelvic drains at admission	1250 ml	1100 ml	1000 ml
Previous interventions to control bleeding (before arrival)	Angiographic embolization & Pelvic packing &	Internal iliac artery ligation & Pelvic packing twice	Internal iliac artery ligation & Pelvic packing twice
No of laparotomies since delivery (before arrival)	1	2	2
Hemoglobin (Hb) concentration (gm%)	8.2 g	9.0 g	8.5 g
Total WBC count (cc)	16.5 X 10^3^	15.2X 10^3^	13.6X10^3^
Platelet count (cc)	108 X10^3^	100 X10^3^	97X10^3^
C-reactive protein (CRP) (mg/dl)	24	48	24
Fibrinogen degradation products (FDP)	Shooting (2500 mg/ml)	normal	normal
Prothrombin concentration	40%	70%	60%

In case 1, pelvic angiographic embolization had been performed at the time of Cesarean subtotal hysterectomy, but this failed to control bleeding completely. She had another laparotomy, which revealed good vascular pedicle ligation and a wide pelvic oozing area from the cervical stump and pelvic floor. Surgical packing was performed, putting in drains, and the patient received a blood transfusion and was then referred to our emergency department. The pelvic surgical drains showed 1250 ml of blood. The very high level of FDP and low prothrombin concentration indicated the presence of coagulopathy.

In case 2, internal iliac artery ligation had been performed at the time of hysterectomy, but this failed to control bleeding. She had another laparotomy, which revealed good vascular pedicle ligation and an oozing of blood from the cervical stump and pelvic floor with no definite source. Surgical packing was performed, putting in surgical drains, and the patient received a blood transfusion. Two days later, she had a second laparotomy to remove the towels, which led to a recurrence of bleeding. Another surgical packing was performed, and then the patient referred to our emergency. The pelvic surgical drains showed 1100 ml of blood.

In case 3, internal iliac artery ligation had been performed at the time of hysterectomy, but this failed to control bleeding. She had a laparotomy, which revealed good vascular pedicle ligation and indefinite wide pelvic venous oozing. Surgical towel packing was performed with the insertion of surgical drains, and the patient received a blood transfusion. Three days later, she had a second laparotomy to remove towels, which led to a recurrence of bleeding. Another surgical packing was performed, followed by referral to our emergency department. The pelvic surgical drains showed 1000 ml of blood.

Pneumatic balloon treatment

**Figure 1:**
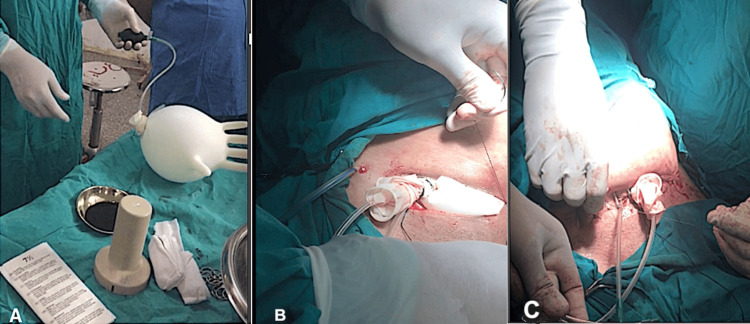
Intraoperative images describing the procedure A: The balloon was made of rubber surgical gloves ligated around a plastic catheter and initially inflated for testing. B: The partially inflated balloon is compressed with a towel for protection during the suturing of the abdominal incision. C: The stem of the catheter and the ligated openings of the gloves were kept just outside the skin incision.

After stabilization of the patients' general conditions and preparation of cross-matched fresh blood, re-laparotomy was performed under general anesthesia to remove the already present surgical towels. When accessing the pelvis, the already present towels were removed one by one. Upon removal of the last deepest towel, pelvic flooding from generalized oozing pelvic side walls and vaginal vault was seen. Another meticulous re-check of ligatures of all vascular pedicles and searching for any non-ligated vessels was done. In all cases, the pelvic tissues were friable and bled with any trials at grasping or suturing. Local hemostatic agents failed to stop bleeding, but it was temporarily arrested as long as towel compression was applied. The pneumatic balloon procedure was performed in three steps (Figures 1A-1C).

Step 1: Balloon Preparation

The balloon was made of two sterile surgical gloves from the same side, with one glove being totally inserted inside the other to form a strong balloon (Figure 1A). The gloves were sterile, rubber/latex-made, of medium size (size 7.5-8.0), normally-stretching, not aged, powder-free, and kept in a good storage environment. A sterile plastic urinary catheter of medium size (14-16) is passed into the hand opening of the inner glove. Then both gloves’ hand openings were fully secured around the catheter stem by a tight silk ligature. The external end of the catheter was connected to a sphygmomanometer's air pump, and an initial glove inflation was done to test for air leaks. Also, the size and the tension of the inflated balloon were initially assessed in relation to its internal pressure, which was measured by attaching the external catheter end to the mercury warehouse of the sphygmomanometer. The balloon was then completely deflated.

Step 2: Balloon Placement and Partial Inflation

The deflated glove balloon was positioned centrally in the pelvis and then partially inflated. The abdomen was closed while the assistant compressed the balloon with a towel into the pelvis for protection, as shown in Figure 1B. The stem of the catheter and the ligated openings of the gloves were kept just outside the skin incision, as shown in Figure 1C. Two abdominal drains were inserted, and a plentiful wound dressing was applied with adhesive compressing plaster. 

Step 3: Abdominal Binder and Full Balloon Inflation

**Figure 2:**
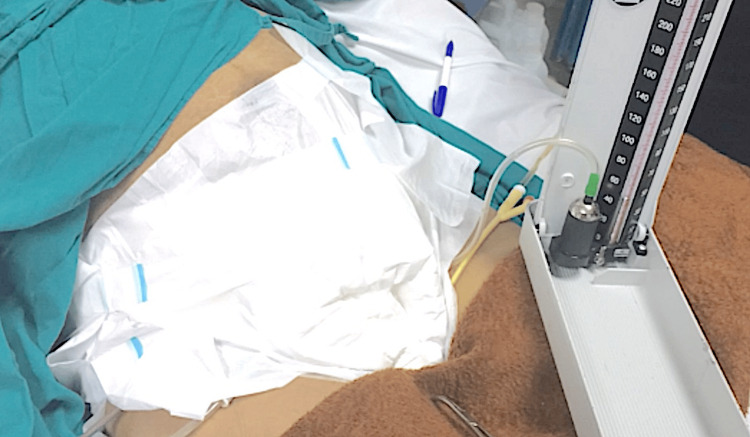
Application of tight external abdominal binder and calibration of the balloon pressure by the sphygmomanometer in the range of 40-60 mmHg

Using three surgical towels and adhesive compressing plaster, an external abdominal binder was applied over and above the skin incision dressing, reaching up to the level of the umbilicus, as shown in Figure 2.

This binder was essential to keep the inflated balloon in situ in the pelvis. The pressure inside the balloon was then increased and calibrated by connecting the outlet of the catheter to the mercury container of a sphygmomanometer, as shown in Figure 2. The pressure used in all cases ranged between 40 and 60 mmHg. The catheter stem was then closed by applying a clamp to maintain the balloon's pressure. 

Postoperative monitoring and removal of the balloon 

A broad-spectrum umbrella of parenteral antibiotics was added. The first and second cases were admitted to the intensive care unit (ICU) in the first 24 hours from balloon application, while on the third case the admission extended to three days. The surgical drains were observed for the amount of blood, and the balloon's pressure was continuously calibrated to keep it constant over the next 24-48 hours, as the initial scheduled duration. A gradual decrease in balloon pressure started 24-48 hours from insertion in the absence of any bleeding. After complete deflation of the balloon, the gloves were retained on site for 24 hours to observe for any bleeding recurrence, then they were removed in a bedside procedure by gentle traction on the catheter stem.

Outcomes

The operative and postoperative data are shown in Table 2.

**Table 2:** Operative and postoperative data of the patients

Data	First case	Second case	Third case
Operative time (Min)	75	40	35
Intraoperative complications	Non	Non	Non
Balloon compression duration (h)	48	24	72
Pressure inside the balloon (mmHg)	50	45	60
Whole duration of balloon retention in the pelvis (h)	72	48	96
Success to stop bleeding	Successful	Successful	Successful
Postoperative complications	Wound infection and gaping	Non	Non
Fresh blood transfusion (units)	4	2	2
Packed red blood cell (units)	3	0	1
Fresh frozen plasma (units)	3	1	4
Hemoglobin concentration on discharge (gm%)	10	10.5	10
Postoperative hospital stays (days)	14	6	7

The bleeding stopped in two cases within the scheduled time of balloon inflation. In the third case, the balloon was re-inflated while in place for an extra 48 hours because of re-bleeding, as evidenced by the blood coming in the drains. The procedure was smoothly performed with decreased operative time from 75 min. in the first case to 35 min. in the last case. The average pressure at which bleeding was stopped ranged between 40 and 60 mmHg. All cases had intraoperative and/or postoperative fresh blood transfusion or fresh frozen plasma in addition to packed red blood cell units to treat anemia. The hospital stay was 14, 6, and 7 days for the first, second, and third cases, respectively. There were no major postoperative complications apart from wound infection in the first case, which required two dry sutures for repair.

## Discussion

In the present case series, it was feasible to perform this simple pelvic pneumatic balloon procedure and control the refractory pelvic hemorrhage with no complications related to the procedure. The possible causes of bleeding in such cases after securing the vascular pedicles are oozing from wide-surface raw areas due to needle puncture sites in coagulopathy, infected friable tissue, and small vessel or venous plexus oozing in inaccessible areas [12]. Pelvic compression is an ancient technique that has the advantage of being able to stop such hemorrhages through persistent mechanical compression of the bleeding blood vessels against the pelvic walls. The efficacy of surgical towel packing in controlling intractable pelvic hemorrhage is in the range of 78% to 100% [5-7]. The inherent complications of this type of compression are the need for a second laparotomy for towel removal and increased risks of febrile morbidity and mortality at a rate of 12.4% [6-8].

The current balloon procedure uses air and mechanical compression instead of fluid-filled balloons. It has multiple advantages: 1) being dynamic for the control of pelvic hemorrhage. In instances of recurrence of bleeding after postoperative initial deflation, re-inflation or increasing pressure while the balloon is in situ can be easily performed. 2) The merits of being less vulnerable to rupture or breaks because of its better tensile strength compared to other methods, such as the condom balloon. 3) Being capacious and able to be inflated to a minimum diameter of 28 cm [13]. 4) The balloon pressure is controllable in objectivity by a sphygmomanometer. 5) Its constituents are always available in the theater and supplied sterile, thus decreasing the risk of febrile morbidity. 6) Its removal is easy and painless as a bedside procedure requiring neither laparotomy nor anesthesia. 7) If pelvic vascular embolization is planned while the balloon is in place, we can partially deflate the tamponade to allow identification of feeding vessels to the bleeding site precisely.

Successful management of such pelvic hemorrhage was previously reported by using fluid-filled balloons. In one case report of post-hysterectomy pelvic hemorrhage, the compression was achieved by a Foley catheter-condom balloon filled with saline [8]. In disagreement with the current series, re-laparotomy 72 h later was performed for removal. In another case series including three patients with post-hysterectomy pelvic hemorrhage, the compression was performed by Bakri's balloon filled with saline [9]. In disagreement with the current case series, the authors passed the inflation port of Bakri's balloon vaginally through posterior culdotomy. They reported control of bleeding when filled with up to 400-550 mL of saline and removed at bedside after 24-30 hours [9]. Bakri's balloon is a 24F, 54-cm-long silicone catheter with a balloon that has a stated optimum capacity of 500 mL of fluid as issued by the manufacturer [9]. In 2006, the FDA approved it for intrauterine placement in the management of atonic postpartum hemorrhage. In our opinion, the Bakri balloon has two limitations for use in such bleeding in the current series. First, the wider oozing pelvic surface area compared to the intrauterine placental site makes it suboptimal to control bleeding unless over-inflated, which carries the risk of rupture. Second, it is unavailable in obstetric theaters most of the time. Also, the Sengstaken-Blakemore tube was described before in a case report of the pelvic floor after hysterectomy due to placenta percreta. The balloon was filled with 470 ml of saline and the tube passed from the cervical stump to outside the vagina with a traction for three days. It succeeded to stop bleeding and then the tube was removed vaginally [14].

## Conclusions

With limited resources, the current novel pneumatic balloon is a noninvasive, feasible, and technically non-demanding procedure when other methods fail. It was found to be a successful lifesaving tool that has a great advantage of removal in a bedside procedure with no major complications. More studies are essential to establish its efficacy and safety as well as the optimum value of the pressure and duration of compression.
